# Constitutive Modeling of the Nonlinear Tensile Response of High-Strength Nanofiber Yarns Under Monotonic Loading

**DOI:** 10.3390/polym18131592

**Published:** 2026-06-26

**Authors:** Qingqing Shao, Jingyu Hu, Qiyu Wei, Jiqiang Cao, Yuanshu Xiao, Xiang Liu, Bo Xing, Xiakeer Saitaer

**Affiliations:** 1 Xinjiang Key Laboratory of Intelligent and Green Textile, College of Textiles & Clothing, Xinjiang University, Urumqi 830046, China; 2 Key Laboratory of Textile Science and Technology (Ministry of Education), College of Textiles, Donghua University, Shanghai 201620, China

**Keywords:** high-strength nanofiber yarns, nonlinear tensile response, constitutive modeling, viscoelasticity, monotonic loading

## Abstract

High-strength nanofiber yarns exhibit pronounced nonlinear tensile responses arising from their hierarchical fibrous architecture, yet compact constitutive descriptions remain limited. Here, high-strength polyacrylonitrile nanofiber yarns were prepared by post-drawing as-spun yarns above the glass transition temperature, and their aligned, stacked morphology was confirmed by scanning electron microscopy. Monotonic tensile tests at different loading rates were used to quantify the rate-dependent stress–strain response. The tangent modulus derived from the tensile curve varied strongly with strain, confirming clear deviation from linear viscoelasticity. To capture this behavior, two effective models were established: a modified nonlinear three-element model and a structural four-element model incorporating a nonlinear elastic contribution. Closed-form stress–strain expressions were derived for constant strain-rate loading and fitted to experimental data using nonlinear regression. Both models reproduced the measured tensile curves with high accuracy over the investigated loading-rate range, with correlation coefficients close to unity and low fitting errors. The identified parameters were highly consistent between formulations, indicating functional equivalence for the present monotonic tensile dataset. These results provide a compact framework for characterizing and designing hierarchical polymer nanofiber yarns.

## 1. Introduction

High-strength nanofiber yarns are emerging as a class of hierarchical fibrous materials that combine low density, structural flexibility, and promising load-bearing capability, making them attractive for advanced textiles, wearable systems, and bioinspired engineering assemblies [1,2,3,4,5]. In contrast to conventional staple or filament yarns, their tensile response is governed not only by the intrinsic deformation of constituent polymer nanofibers, but also by collective mechanisms associated with filament alignment, packing compactness, inter-fiber contact, and stress redistribution within the yarn architecture [1,2,3,5]. Owing to this multilevel structural organization, the macroscopic tensile behavior of nanofiber yarns often deviates from the assumptions of linear elasticity or linear viscoelasticity, especially when high orientation and structural densification are introduced during post-processing [6,7,8,9,10].

Considerable efforts have therefore been devoted to the fabrication and structural optimization of electrospun nanofiber yarns. Early studies demonstrated that aligned and twisted polyacrylonitrile nanofibers could be assembled into continuous yarns with significantly improved tensile properties compared with randomly deposited nanofiber mats [1]. Subsequent work further showed that post-spinning stretching is highly effective in enhancing molecular orientation, crystalline ordering, and load transfer efficiency, thereby markedly increasing the stiffness and strength of electrospun polyacrylonitrile nanofibers and yarn-like assemblies [6,7]. In parallel, continuous fabrication strategies for aligned nanofiber yarns and micro-yarns have been developed, and the effects of twist, ionic mediation, and structural tailoring on yarn mechanical performance have been systematically discussed [8,9]. More recent studies have extended the design of nanofiber yarns toward smart and functional systems, indicating that microstructure tailoring remains a key route to simultaneously tuning strength, deformability, and multifunctionality [4,5,10]. These studies clearly establish that the tensile response of nanofiber yarns is strongly structure-dependent; however, most existing efforts remain focused on fabrication, morphology evolution, and property enhancement, whereas compact constitutive descriptions of their nonlinear tensile response remain relatively limited.

From a mechanics perspective, constitutive modeling is essential for understanding and predicting the deformation behavior of fibrous materials under loading. Classical spring–dashpot rheological models have long been used to describe the time-dependent response of yarns, fibers, ropes, and related slender assemblies [11,12,13,14,15]. For textile yarns, relaxation and creep behavior have been analyzed using generalized Maxwell-type representations and mechanical analog models, which provide convenient routes for parameter identification and engineering interpretation [13,14]. For highly oriented polymeric fibers and yarns, nonlinear viscoelastic and viscoelastic–viscoplastic extensions have also been proposed to account for strain-dependent deformation and irreversible contributions under loading [11,12]. Similar constitutive ideas have been successfully used in the analysis of synthetic fiber ropes, where rate dependence, delayed elasticity, and nonlinear recovery behavior must be represented within a relatively compact framework [15]. These studies confirm that rheological modeling remains a powerful tool for fibrous systems, yet they also suggest that model selection becomes challenging when the material exhibits pronounced nonlinear tensile behavior arising from hierarchical structural effects.

In recent years, constitutive formulations for hierarchical fibrous materials have been further developed in adjacent fields. For example, nonlinear Maxwell-type descriptions have been used to capture the tensile response of collagen fibrils [16], while viscoelastic-plastic and visco-hyperelastic models have been introduced to describe the compaction and rate-dependent behavior of dry fiber fabrics and woven architectures [17,18,19]. Likewise, nonlinear viscoelastic–viscoplastic models have been reported for other polymer-based material systems exhibiting coupled elastic, viscous, and irreversible deformation mechanisms [20]. These advances indicate that compact constitutive models can effectively represent complex mechanical responses in structurally organized materials. Nevertheless, for high-strength nanofiber yarns subjected to monotonic tension, there is still a lack of clear constitutive formulations that are both simple enough for robust identification and sufficiently expressive to capture the observed nonlinear stress–strain response. In particular, the relationship and practical equivalence between alternative rheological formulations for such yarns have not been sufficiently clarified.

The present work addresses this issue by investigating the nonlinear tensile response of high-strength polyacrylonitrile nanofiber yarns obtained through post-drawing treatment. Based on the experimentally observed evolution of the tensile curve, two effective constitutive formulations are established: a modified nonlinear three-element model and a structural four-element model incorporating a nonlinear elastic contribution. Closed-form stress–strain expressions are derived under constant strain-rate monotonic loading, and the model parameters are identified using tensile data obtained at different loading rates. The objectives of this study are threefold: first, to establish compact constitutive equations capable of reproducing the nonlinear tensile response of high-strength nanofiber yarns; second, to evaluate the robustness of the proposed formulations across loading rates; and third, to assess whether the two constitutive models exhibit functional equivalence for the present monotonic tensile dataset. By doing so, this work provides a mechanics-oriented framework for the constitutive characterization of hierarchical nanofiber yarns and offers guidance for simplified model selection in related fibrous material systems.

## 2. Materials and Methods

### 2.1. Materials

N,N′-dimethylformamide (DMF, 99.99%) was obtained from Fisher Chemical (Waltham, MA, USA). Polyacrylonitrile (PAN, Mn of 120,000, co-polymer with 6 wt% methyl acrylate) was supplied by Dolan GmbH (Kelheim, Germany). Acetone and ethanol (technical grade, Bayreuth, Germany) were purified by distillation.

### 2.2. Preparation of High-Strength Nanofiber Yarns

**Figure 1:**
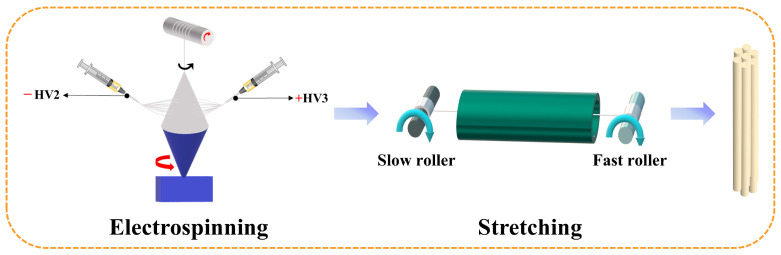
Schematic diagram of the preparation of high-strength nanofiber yarn [21].

The PAN nanofiber yarns used in this study were fabricated following our previously reported electrospinning–hot stretching method [21]. Briefly, the PAN spinning solution was supplied to the electrospinning system, and continuous PAN nanofibers were generated under the applied electrostatic field. In Figure 1, HV2 and HV3 denote the high-voltage power supplies/electrodes used to establish the electric field during electrospinning. The electrospun nanofibers were continuously collected and assembled into as-spun PAN nanofiber yarns. Subsequently, the as-spun yarns were stretched along the yarn axis at 160 °C, which is above the glass transition temperature of PAN [22]. The stretching treatment was performed using a roll-to-roll thermal drawing process. During this process, the yarns passed successively through a slow roller and a fast roller, and the drawing deformation was mainly generated by the speed difference between the two rollers rather than by an additional external compression pressure. This thermal stretching process promoted nanofiber alignment along the yarn axis, improved the compactness of the yarn structure, and consequently enhanced the mechanical strength of the yarns. Further details regarding the spinning setup, stretching procedure, and structural characterization are available in reference [21].

### 2.3. Characterization

The morphology of the yarns was obtained through SEM testing. Prior to acquiring the cross-sectional SEM images, the samples were soaked in ethanol and water for 0.5 h, and then cut in liquid nitrogen. The mechanical properties of the yarns were tested using a material testing machine (zwickiLine Z0.5, BT1-FR0.5TN.D14, Zwick/Roell, Ulm, Germany). The tensile test was conducted as a longitudinal uniaxial stretching test along the yarn axis. Both ends of the PAN nanofiber yarn were fixed by the upper and lower grips of the testing machine, and the yarn was stretched in the axial direction under a single-extension program until fracture. The testing parameters were as follows: the grip spacing was set at 10 mm, the crosshead extension rates were set at 1, 5, and 10 mm/min, the preload was 0.005 cN, and the temperature was maintained at 25 °C. The load cell used was the Zwick/Roell KAF TC, with a nominal load of 20 N. The tensile stress–strain curves were recorded during the test. For each loading condition, three independent yarn specimens were tested to confirm the repeatability of the tensile response. For the representative tensile condition at 5 mm/min, the main tensile parameters were reported as mean ± standard deviation. Representative curves with consistent tensile behavior were selected for the subsequent model fitting and analysis.

## 3. Results and Discussion

**Figure 2:**
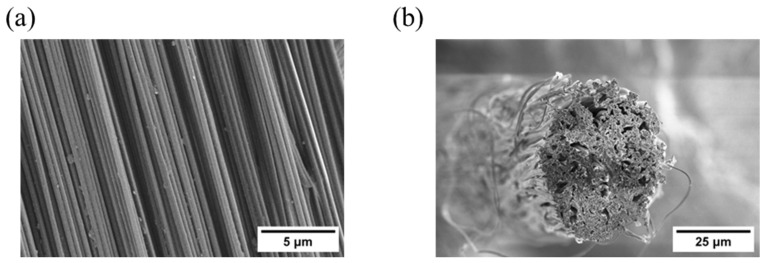
SEM images of high-strength PAN nanofiber yarn: (**a**) longitudinal morphology showing aligned nanofibers along the yarn axis; (**b**) cross-sectional morphology showing the stacked fibrous structure.

Figure 2 shows that the high-strength nanofiber yarn is composed of multiple nanofibers that are generally aligned along the yarn axis. To further support this observation and verify the structural assumption used in the subsequent mechanical modeling, a simple image-based orientation evaluation was performed from representative SEM images. The yarn axis was defined as 0°, and the deviation angle between visible fiber segments and the yarn axis was measured. The average deviation angle was approximately 3.5°. Using the two-dimensional orientation parameter [23], S = 2<cos^2^θ> − 1, where θ is the deviation angle between the fiber segment and the yarn axis, the estimated orientation factor was approximately 0.99. This result confirms that the nanofibers were highly oriented along the yarn axis and supports the simplifying assumption that the nanofibers can be regarded as approximately parallel to the yarn axis in the structural mechanical model.

The cross-sectional image further confirms the stacked structure of the yarn (Figure 2b). Therefore, the following assumptions are made regarding the stacking structure of the yarn: (i) the high-strength nanofiber yarn is composed of uniform nanofibers with the same diameter and (ii) the nanofibers are arranged approximately parallel to the yarn axis, neglecting any interruptions in the nanofibers. This stacking structure of the high-strength nanofiber yarn provides a basis for the subsequent establishment of the structural mechanical model.

### 3.1. Tensile Characterization of High-Strength Nanofiber Yarn

**Figure 3:**
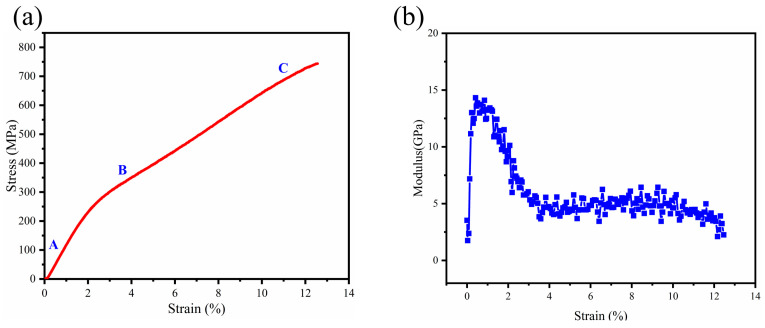
Tensile behavior of high-strength PAN nanofiber yarn: (**a**) typical monotonic stress–strain curve with three deformation stages; (**b**) tangent modulus–strain curve obtained from the first derivative of the smoothed stress–strain data.

The tensile stress–strain curve of the high-strength nanofiber yarn exhibits a pronounced nonlinear stress–strain response, with an initial concave region followed by a convex response, reflecting the complex deformation mechanisms of the hierarchical yarn structure during tensile loading (Figure 3a). Based on three independent specimens tested at 5 mm/min, the elongation at break and tensile strength of the high-strength nanofiber yarns were 12.539 ± 0.025% and 743.370 ± 0.400 MPa, respectively. The low standard deviations indicate acceptable repeatability of the tensile measurements. The tensile process can be divided into three stages. In the initial stage (A), the concave stress–strain response should not be simply interpreted as linear elastic deformation. Based on the SEM-observed aligned and stacked nanofiber structure and the rapid increase in tangent modulus at low strain, this response may be associated with initial structural adjustment and viscoelastic deformation of the yarn, including the tightening of loosely packed nanofiber segments, minor adjustment of fiber orientation, and progressive establishment of load transfer among nanofibers. It should be noted that this interpretation is inferred from the mechanical response and SEM-observed morphology, rather than directly confirmed by in situ tensile observation. In the intermediate stage (B), the curve shows a pronounced nonlinear convex feature, indicating that the yarn enters a nonlinear deformation region. During this stage, the stress increases rapidly with increasing strain, suggesting possible further fiber tightening or alignment, improved inter-fiber interaction, and more effective stress transfer along the yarn axis. In the final stage (C), the curve gradually tends to flatten before fracture, suggesting possible fiber slippage, structural rearrangement, and local damage effects. However, because post-fracture SEM characterization and in situ tensile observation were not performed in this study, specific microscopic failure modes, such as fiber pull-out, fiber breaking, or fiber bundling, cannot be directly identified and are not explicitly assigned here. It should also be noted that these damage-related effects are discussed as a qualitative interpretation of the monotonic tensile response and are not explicitly modeled by an independent damage variable in the present constitutive framework. Although the stress continues to increase, the rate of increase becomes lower, indicating a stabilized large-deformation stage prior to failure.

To further clarify the nonlinear tensile behavior of the high-strength nanofiber yarn, the first derivative of the stress–strain curve was calculated to obtain the corresponding modulus–strain curve, as shown in Figure 3b. Before differentiation, the raw stress–strain data were smoothed to reduce high-frequency experimental noise. The tangent modulus was then calculated as dσ/dε using a finite-difference method. The noisy data points associated with the initial gripping adjustment and the abrupt stress drop at final fracture were excluded from the derivative calculation. This procedure reduced noise amplification during numerical differentiation while preserving the overall evolution trend of the modulus–strain curve. In the initial low-strain range of 0–1%, the modulus increases rapidly and reaches a peak, which can be attributed to the rapid tightening and structural adjustment of the yarn under the initial tensile load. With further increasing strain, the modulus gradually decreases and then tends to stabilize at higher strains. This evolution indicates that the yarn undergoes a transition from initial structural adjustment to nonlinear viscoelastic deformation and then to a relatively stable large-deformation stage. The reduction and stabilization of the modulus at large strains suggest that, despite substantial deformation, the yarn can still maintain a certain load-bearing capacity. These results demonstrate that the high-strength nanofiber yarn possesses excellent mechanical properties under large-strain conditions, combining high strength with good toughness.

### 3.2. Theoretical Mechanical Modeling of the Nonlinear Viscoelastic Mechanical Behavior of High-Strength Nanofiber Yarns

(1) Nonlinear spring three-element model

The three-element model, consisting of two Hookean springs and a Newtonian dashpot, is frequently used to describe the viscoelastic phenomena of textile polymers. It effectively characterizes the viscoelastic mechanical properties of textiles under small deformation conditions. The primary assumption of the model is that the material exhibits linear viscoelasticity, meaning that the relationship between stress and strain can be described by linear differential equations, and the deformation remains within the small deformation range. However, the viscoelastic behavior of real polymer materials does not fully conform to the assumptions of the three-element model. Because the deformations in textiles are relatively large and essentially nonlinear, it is often observed that under a given stress, textiles exhibit linear viscoelastic behavior in the short term, but this behavior becomes nonlinear over time.

**Figure 4:**
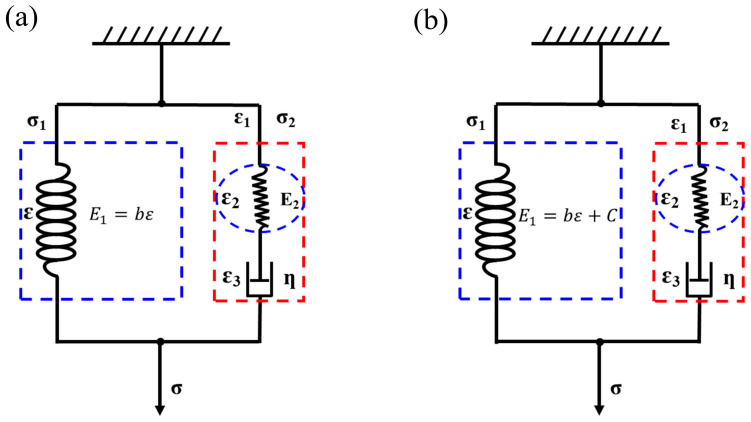
Nonlinear three-element models: (**a**) original Vangheluwe model; (**b**) modified Vangheluwe model with an initial modulus C.

In 1992, Vangheluwe [24] proposed replacing the Hookean springs with nonlinear springs, which better describe the tensile mechanical behavior of fiber bundles or yarns, as shown in Figure 4a. The model is based on the following assumptions: First, the material is assumed to exhibit linear elasticity within a certain stress range, where a linear relationship exists between stress and strain. This assumption applies to the initial loading stage of fiber bundles or yarns. Second, it is assumed that the fibers within the fiber bundle are arranged in parallel and subjected to force simultaneously during loading, neglecting friction and interactions between fibers. It should be noted that this assumption does not imply that inter-fiber interactions are physically absent. Instead, individual fiber–fiber friction, contact sliding, and local stress redistribution are not explicitly resolved at the single-fiber level, but are incorporated into the effective nonlinear elastic and viscous parameters of the yarn-level constitutive model. Additionally, it is assumed that the stress distribution within the fiber bundle is uniform, and all fibers share the external stress equally during loading. Furthermore, the material is assumed to be homogeneous, meaning that each part of the material has identical physical and mechanical properties, thereby simplifying mathematical treatment. It is also assumed that during tensile loading, some fibers in the bundle will gradually fracture, and this fracture occurs randomly, following a specific statistical distribution (e.g., Weibull distribution) to describe the behavior of the fiber bundle at high strain stages. These assumptions allow the Vangheluwe model to effectively describe the tensile mechanical properties of fiber materials.

Based on the tensile stress–strain curve shown in Figure 3a, the high-strength nanofiber yarn is treated as an effective homogenized yarn-level viscoelastic system for constitutive modeling. This assumption does not imply that the yarn is perfectly homogeneous or free of voids. As observed in the SEM cross-sectional images, local gaps and heterogeneous fiber packing may exist within the yarn. In the present compact model, these local structural features, together with inter-fiber contact and packing effects, are not explicitly resolved at the single-fiber or pore level. Instead, their collective influence is incorporated into the effective constitutive parameters of the model, including the nonlinear elastic contribution and viscous resistance. The nonlinear spring three-element (Vangheluwe) model was chosen to characterize its viscoelastic mechanical behavior. In the Vangheluwe model, when the strain is zero, the modulus of the nonlinear spring is also zero, which does not match the actual behavior (Figure 4a). Therefore, the model was modified, as shown in Figure 4b. Let C be the initial modulus of the nonlinear spring. The modulus of the nonlinear spring is $E_{1}=b\varepsilon +C$, and the spring in the Maxwell model parallel to the nonlinear spring is a Hookean spring.

From the above Figure 4, it can be seen that the only difference between models (a) and (b) lies in the initial modulus of the nonlinear spring. In model (a), the modulus C is always equal to 0 (C = 0), while in model (b), C is a constant greater than 0 (C > 0). In the diagram: σ_1_, σ_2_, and σ represent stress; $\varepsilon_{1}$, $\varepsilon_{2}$, and ε represent strain; $E_{1}$ is the modulus of the nonlinear spring; $E_{2}$ is the modulus of the Hookean spring; η is the viscosity coefficient of the Newtonian dashpot; and C is the initial modulus of the nonlinear spring, with b being the coefficient of the nonlinear spring.

As shown in Figure 4b, the model consists of a nonlinear spring (on the left) in parallel with a combination of a linear spring and a Newtonian dashpot in series (on the right). Therefore, in this model, the strain on both sides is equal, and the total stress is the sum of the stresses on both sides. The intrinsic relationship equations are as follows:

(1) $$\sigma =\sigma_{1}+\sigma_{2}$$

(2) $$\varepsilon =\varepsilon_{1}$$

The stress–strain relationship of the nonlinear spring on the left side of the model is:

(3) $$\sigma_{1}=(b\varepsilon +C)\varepsilon$$

The right-hand side of the model is essentially a Maxwell model, where the total strain is the sum of the strains in the Hookean spring and the Newtonian dashpot [25]. The stresses in both components are equal:

(4) $$\varepsilon_{1}=\varepsilon_{2}+\varepsilon_{3}$$

(5) $$\sigma_{2}=E_{2}\varepsilon_{2}=\eta \frac{d\varepsilon_{3}}{dt}$$

By combining Equations (2) and (4), we obtain:

(6) $$\varepsilon_{3}=\varepsilon -\varepsilon_{2}$$

Substituting this into Equation (5), we get:

(7) $$\eta \frac{d\varepsilon_{2}}{dt}+E_{2}\varepsilon_{2}=\eta \frac{d\varepsilon }{dt}$$

Substituting Equation (3) into Equation (1), we get:

(8) $$\sigma =(b\varepsilon +C)\varepsilon +\sigma_{2}$$

After rearranging, the constitutive equation of the model is:

(9) $$\eta \dot{\varepsilon }+(\frac{2b\eta }{E_{2}}+b\varepsilon +c)\varepsilon +\frac{\eta c}{E_{2}}=\frac{\eta }{E_{2}}\dot{\sigma }+\sigma$$

In the mechanical performance tests in this paper, all yarns were stretched at a constant rate:

(10) $$\frac{d\varepsilon }{dt}=k$$

Solving Equation (9) gives the equation for the modified Vangheluwe model:

(11) $$\sigma (\varepsilon )=\eta k(1-e^{-\frac{E_{2}}{\eta k}\varepsilon })+(b\varepsilon +C)\varepsilon$$

(2) Structural four-element model

As shown in Figure 3, the high-strength nanofiber yarn exhibits typical nonlinear viscoelastic behavior. In addition to instantaneous elasticity and delayed elasticity, the deformation of the yarn also involves irreversible plastic deformation due to the slippage between molecular chain segments. Therefore, a four-element structural mechanical model is further established.

**Figure 5:**
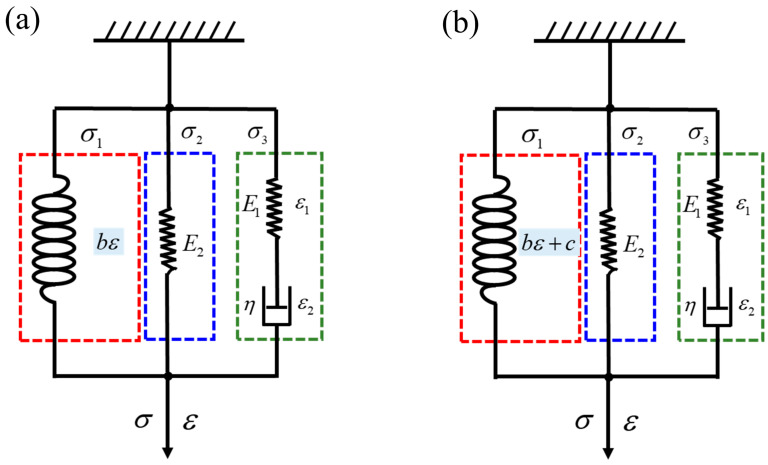
Structural four-element models for high-strength PAN nanofiber yarn: (**a**) model without an initial nonlinear-spring modulus; (**b**) model with an initial nonlinear-spring modulus C. The spring and dashpot elements represent the effective elastic and viscous responses of the yarn.

From the stacking structure of high-strength nanofiber yarn, it can be seen that the nanofibers can be considered as a standard linear solid model, which consists of two Hookean springs and a Newtonian dashpot. The standard linear solid model can be expressed in different but mechanically equivalent arrangements, including the Maxwell form and the Kelvin form [25]. In the Maxwell form, the model consists of a Maxwell element, namely a Hookean spring in series with a Newtonian dashpot, arranged in parallel with an additional Hookean spring [25,26]. For computational convenience and consistency with the following derivation, the Maxwell form of the standard linear solid model is adopted in this study. The contact layers between fibers are represented using nonlinear springs. The structural four-element model for characterizing the tensile mechanical behavior of high-strength nanofiber yarn should consist of the Maxwell element, Hookean spring, and nonlinear spring in parallel, forming a four-element model of the standard linear solid model in conjunction with a nonlinear spring, as shown in Figure 5.

In the model (a) shown in Figure 5a, the initial modulus of the nonlinear spring is 0. To align the initial modulus of the nonlinear spring with actual conditions, its initial modulus is set as C in model (b). From Figure 5a, it can be seen that this model consists of a nonlinear spring (on the left), a Hookean spring (in the middle), and a Maxwell model (on the right) in parallel. Therefore, in this model, the strains of the three components are equal, and the total stress is the sum of the stresses from the three components. Based on the characteristics of the mechanical model, the total strain of the yarn is the sum of the strains in the Hookean spring (E_1_) and the Newtonian dashpot, that is:

(12) $$\varepsilon =\varepsilon_{1}+\varepsilon_{2}$$

The total stress of the yarn is:

(13) $$\sigma =\sigma_{1}+\sigma_{2}+\sigma_{3}$$

The relationship between stress and strain in the left-side nonlinear spring is:

(14) $$\sigma_{1}=b\varepsilon^{2}$$

The relationship between stress and strain in the middle Hookean spring is:

(15) $$\sigma_{2}=E_{2}\varepsilon$$

In the right-side Maxwell model, according to the deformation characteristics of the model, the stress in the spring and the Newtonian dashpot is equal to the total stress, i.e.:

(16) $$\sigma_{3}=E_{1}\varepsilon_{1}=\eta \frac{d\varepsilon_{2}}{dt}$$

Based on the combination relationship between the Hookean spring and the Newtonian dashpot in the Maxwell model shown in Figure 5a, the corresponding differential constitutive relationship for this model can be derived as:

(17) $$\begin{array}{l} \frac{d\varepsilon }{dt}=\frac{d\varepsilon_{1}}{dt}+\frac{d\varepsilon_{2}}{dt}=\frac{1}{E_{1}}\frac{d\sigma_{3}}{dt}+\frac{\sigma_{3}}{\eta }\\ =\frac{1}{E_{1}}(\frac{d\sigma }{\mathrm{dt}}-2b\varepsilon \frac{d\varepsilon }{\mathrm{dt}}-E_{2}\frac{d\varepsilon }{\mathrm{dt}})+\frac{\sigma -b\varepsilon^{2}-E_{2}\varepsilon }{\eta } \end{array}$$

In the mechanical performance tests in this paper, the tensile loading of the yarn is at a constant rate, represented as *k*, thus:

(18) $$k=\frac{d\varepsilon }{dt}$$

Substituting Equation (18) into Equation (17), we derive the relationship between stress and strain for this model:

(19) $$\frac{d\sigma }{\mathrm{dt}}+\frac{E_{1}}{\eta }\sigma =\left(E_{1}+E_{2}+2b\varepsilon \right)\frac{d\varepsilon }{\mathrm{dt}}+\frac{E_{1}E_{2}}{\eta }\varepsilon +\frac{E_{1}b}{\eta }\varepsilon^{2}$$

Further simplifying yields:

(20) $$\frac{d\sigma }{\mathrm{dt}}+\frac{E_{1}}{\eta }\sigma =\frac{bE_{1}}{\eta }k^{2}t^{2}+2bk^{2}t+\frac{E_{1}E_{2}}{\eta }kt+E_{1}k+E_{2}k$$

Solving the above differential equation gives:

(21) $$\sigma =C_{1}e^{-\frac{E_{1}}{\eta }t}+bk^{2}t^{2}+E_{2}kt+\eta k$$

When t = 0, σ = 0; thus, substituting Equation (18) into Equation (21) provides the stress–strain relationship for the nanofiber yarn:

(22) $$\sigma \left(\varepsilon \right)=\eta k\left(1-e^{-\frac{E_{1}\varepsilon }{\eta k}}\right)+b\varepsilon^{2}+E_{2}\varepsilon$$

In model (b) shown in Figure 5, the relationship between stress and strain in the left-side nonlinear spring is:

(23) $$\sigma_{1}=\left(b\varepsilon +C\right)\varepsilon =b\varepsilon^{2}+C\varepsilon$$

The derivation for the portion shown in model (b) is similar to that of model (a), and thus will not be repeated here. Similarly, the constitutive relationship is:

(24) $$\begin{array}{l} \frac{d\varepsilon }{dt}=\frac{d\varepsilon_{1}}{dt}+\frac{d\varepsilon_{2}}{dt}\\ =\frac{1}{E_{1}}(\frac{d\sigma }{\mathrm{dt}}-2b\varepsilon \frac{d\varepsilon }{\mathrm{dt}}-C\frac{d\varepsilon }{\mathrm{dt}}-E_{2}\frac{d\varepsilon }{\mathrm{dt}})+\frac{\sigma -b\varepsilon^{2}-C\varepsilon -E_{2}\varepsilon }{\eta } \end{array}$$

Rearranging yields:

(25) $$\frac{d\sigma }{\mathrm{dt}}+\frac{E_{1}}{\eta }\sigma =\frac{bE_{1}}{\eta }k^{2}t^{2}+2bk^{2}t+\frac{CE_{1}}{\eta }kt+\frac{E_{1}E_{2}}{\eta }kt+Ck+E_{1}k+E_{2}k$$

Solving the above differential equation yields:

(26) $$\sigma =C_{1}e^{-\frac{E_{1}}{\eta }t}+bk^{2}t^{2}+Ckt+E_{2}kt+\eta k$$

When t=0, σ=0, solving $C_{1}=-\eta k$, substituting (18) into (26), we can obtain the stress equation:

(27) $$\sigma \left(\varepsilon \right)=\eta k\left(1-e^{-\frac{E_{1}\varepsilon }{\eta k}}\right)+b\varepsilon^{2}+\left(E_{2}+C\right)\varepsilon$$

### 3.3. Optimization and Experimental Evaluation of Mechanical Model

To select the optimal mechanical model, three widely used statistical indicators were chosen: the correlation coefficient (R), the coefficient of determination (R^2^), and the root mean square error (RMSE). The R measures the strength of the linear relationship between the predicted values and the actual observed values; the R^2^ indicates the proportion of variance in the actual values that is explained by the model; and the RMSE represents the error between the predicted values and the actual observed values. A correlation coefficient absolute value closer to 1, a coefficient of determination closer to 1, and a smaller root mean square error indicate a better fit of the model.

(28) $$R=\frac{\sum_{i=1}^{n}(y_{i}-\bar{y})({\hat{y}}_{i}-\bar{\hat{y}})}{\sqrt{\sum_{i=1}^{n}{\left(y_{i}-\bar{y}\right)}^{2}\sum_{i=1}^{n}{\left({\hat{y}}_{i}-\bar{\hat{y}}\right)}^{2}}}$$

(29) $$R^{2}=1-\frac{\sum_{i=1}^{n}{\left(y_{i}-{\hat{y}}_{i}\right)}^{2}}{\sum_{i=1}^{n}{\left(y_{i}-\bar{y}\right)}^{2}}$$

(30) $$RMSE=\sqrt{\frac{1}{n}\sum_{i=1}^{n}{\left(y_{i}-{\hat{y}}_{i}\right)}^{2}}$$

In the equations, y_i_ represents the measured values; $\bar{y}$ represents the average of the measured values; ${\hat{y}}_{i}$ represents the predicted values; $\bar{\hat{y}}$ represents the average of the predicted values; and n represents the number of samples.

For the three aforementioned viscoelastic mechanical models, the Levenberg–Marquardt optimization algorithm in Origin software was used to perform nonlinear fitting of the tensile experimental data, allowing for comparison and selection of the appropriate tensile mechanical model. For clarity, the modified nonlinear three-element model, the structural four-element model without an initial nonlinear-spring modulus, and the structural four-element model with an initial nonlinear-spring modulus are denoted as Model 1, Model 2, and Model 3, respectively. The measured tensile results are referred to as experimental data.

**Figure 6:**
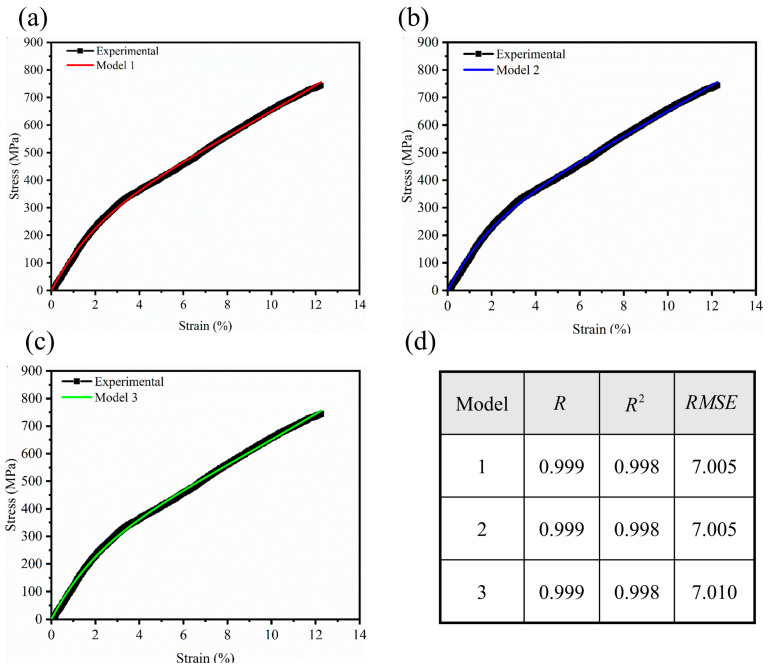
Fitting comparison between experimental data and the three viscoelastic models at k = 5 min^−1^: (**a**) Model 1; (**b**) Model 2; (**c**) Model 3; (**d**) goodness-of-fit indicators, including R, R^2^, and RMSE.

Figure 6 shows the theoretical and experimental stress–strain curves for the three viscoelastic mechanical models of high-strength nanofiber yarn. The results indicate that the theoretical curves of all three models closely match the experimental data. Both the correlation coefficient and the coefficient of determination are close to 1, at 0.999 and 0.998, respectively, demonstrating that the models can accurately fit the current experimental stress–strain data. However, high R^2^ values alone are not sufficient to demonstrate model reliability or exclude the possibility of overfitting, especially for models with multiple adjustable parameters. Therefore, model comparison was not based solely on R^2^. Model 3, the structural four-element model (b), has a root mean square error of 7.010, which is greater than those of the other two models (7.005). This discrepancy may be related to possible parameter interdependence and overparameterization in Model 3, which can reduce convergence stability during nonlinear fitting. Since a formal parameter correlation matrix or sensitivity analysis was not performed in the present work, overparameterization is considered here as a possible explanation rather than a statistically confirmed conclusion. Therefore, convergence stability, RMSE, parameter consistency, and physical interpretability were also considered in the model selection. Based on these considerations, Model 1 and Model 2 show better fitting stability and parameter reliability. It should also be noted that the fitting results represent the goodness of fit for the current experimental dataset, rather than independent external validation of predictive capability. Since an independent external dataset was not used in the present study, the predictive capability and generalizability of the proposed models require further validation using independent yarn batches, processing conditions, polymer systems, or loading regimes. In summary, the modified nonlinear three-element model and the structural four-element model (a) provide reliable and physically interpretable descriptions of the monotonic tensile response of high-strength nanofiber yarn within the investigated experimental range.

**Figure 7:**
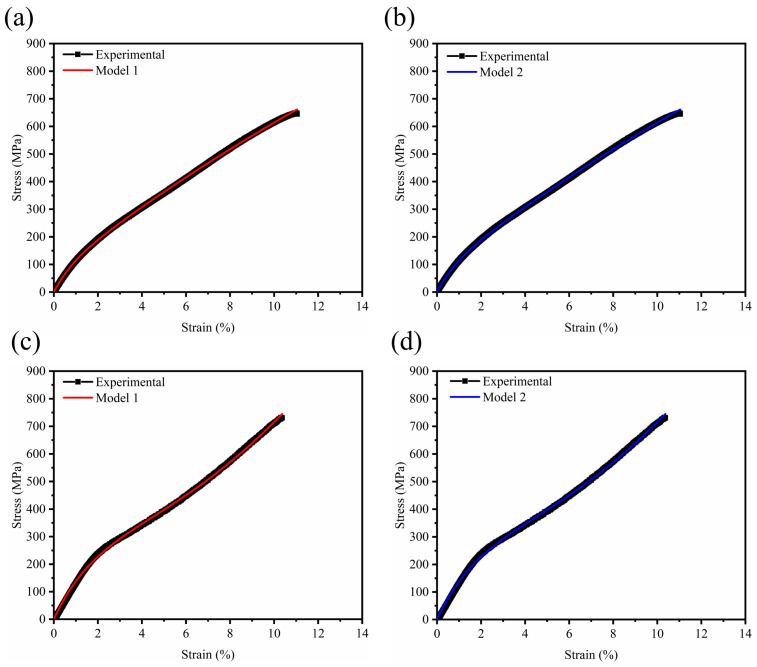
Fitting comparison between experimental data and the two selected models at different loading rates: (**a**) Model 1 at k = 1 min^−1^; (**b**) Model 2 at k = 1 min^−1^; (**c**) Model 1 at k = 10 min^−1^; (**d**) Model 2 at k = 10 min^−1^.

To verify the reliability of the two selected models under different strain rate conditions, the experimental tensile curves of high-strength nanofiber yarn at strain rates of 1/min and 10/min were fitted. The results are shown in Figure 7 and Table 1. At the lower strain rate (1/min), the correlation coefficient and the coefficient of determination for both models are closer to 1, at 0.9998 and 0.9997, respectively, while the RMSE is relatively small, at only 3.1178. This indicates that the models exhibit higher fitting accuracy at low strain rates.

In contrast, at the higher strain rate (10/min), although the correlation coefficient and coefficient of determination remain close to 1 (0.9996 and 0.9991), they are slightly lower than those at the low strain rate, and the RMSE increases to 6.0370. This suggests that the fitting accuracy of the models is higher at low strain rates, allowing for a more precise description of the yarn’s mechanical behavior. At high strain rates, the stress behavior of the yarn becomes more complex, potentially due to factors such as dynamic stress relaxation within the fibers, slippage of molecular chains, and changes in alignment, leading to a decrease in fitting accuracy. The loading speeds of 1, 5, and 10 mm/min were selected based on the structural characteristics of the PAN nanofiber yarns. Unlike conventional textile yarns, the present nanofiber yarns are composed of stacked and aligned nanoscale fibers and have a relatively small specimen size and limited absolute load-bearing capacity. Therefore, relatively mild quasi-static loading conditions were adopted to maintain stable gripping and continuous tensile deformation while avoiding specimen slippage, stress concentration near the grips, or premature fracture. Within this investigated loading range, both selected models showed high fitting accuracy. However, the increased RMSE at the higher loading speed indicates that the fitted parameters should not be directly extrapolated to much lower or much higher strain-rate conditions without further validation.

Based on the above analysis, the theoretical fitting expressions for the two selected mechanical models at low strain rates are as follows.

Nonlinear Spring Three-Element Model (Model 1):

(31) $$\sigma (\varepsilon )=77.09(1-e^{-1.23\varepsilon })+(-0.69\varepsilon +60.50)\varepsilon$$

Structural Four-Element Model (Model 2):

(32) $$\sigma \left(\varepsilon \right)=77.09\left(1-e^{-1.23\varepsilon }\right)-0.69\varepsilon^{2}+60.50\varepsilon$$

The parameters of Model 1 and Model 2 were obtained by nonlinear fitting of the experimental tensile data, and the fitted results are listed in Table 2.

The parameters listed in Table 2 have been mathematically defined in the model derivation above. Here, they are further interpreted as effective yarn-level parameters related to the hierarchical structure of the nanofiber yarn. The viscosity coefficient η reflects the effective time-dependent resistance during tensile deformation, which may be associated with molecular chain mobility, internal friction, inter-fiber sliding, and structural rearrangement. The elastic parameters E_1_ and E_2_ represent effective stiffness contributions related to the aligned nanofiber network, molecular orientation induced by hot stretching, yarn compactness, and inter-fiber stress transfer. The parameter C represents the initial stiffness contribution of the nonlinear elastic component, while b describes the strain-dependent evolution of this nonlinear contribution. Therefore, these fitted parameters should be regarded as effective constitutive parameters reflecting the combined influence of fiber alignment, molecular orientation, inter-fiber interactions, and yarn architecture, rather than direct measurements of individual microstructural features.

The parameters calculated for Model 1 and Model 2 are almost identical, indicating that the modified nonlinear three-element model and the structural four-element model provide functionally similar and nearly indistinguishable predictions under the investigated quasi-static monotonic tensile conditions. This similarity does not mean that the two rheological structures are universally equivalent. Rather, it suggests that, for the present dataset, both models contain the dominant mechanical contributions required to reproduce the observed tensile response, namely an effective viscoelastic contribution and a nonlinear elastic contribution. Model 1 provides a compact phenomenological description and is sufficient for simplified stress–strain fitting and parameter identification under the investigated conditions. In contrast, Model 2 provides a more structure-related interpretation by explicitly considering the standard linear solid component and the nonlinear elastic contribution associated with inter-fiber contact and structural rearrangement. Therefore, the agreement between the two models indicates that the dominant monotonic tensile response of the yarn can be effectively represented by a simplified constitutive form without obvious loss of fitting accuracy. However, this agreement should be regarded as specific to the current dataset and loading condition. Under cyclic loading, creep, stress relaxation, higher strain-rate loading, or temperature-dependent deformation, the two rheological structures may exhibit different predictive behavior and require further validation.

To further highlight the merit of the present approach, a concise comparison between the proposed models and representative constitutive models reported in the literature is provided in Table 3. Since constitutive parameters are highly dependent on material type, specimen geometry, loading mode, strain rate, and parameter definitions, direct numerical comparison across different studies may not be fully meaningful. Therefore, Table 3 mainly compares the model structure and parameter characteristics.

Compared with the representative constitutive models listed in Table 3, the present approach uses a relatively compact set of physically interpretable parameters while maintaining high fitting accuracy for the nonlinear monotonic tensile response of high-strength PAN nanofiber yarns. This comparison further highlights the applicability of the proposed models for simplified parameter identification and mechanical response prediction of hierarchical nanofiber yarns under monotonic loading.

## 4. Conclusions

A modified nonlinear three-element model and a structural four-element model were developed to characterize the nonlinear tensile response of high-strength PAN nanofiber yarns under monotonic loading. The experimental results confirmed a clear departure from linear viscoelasticity, indicating that the tensile behavior of the yarns is governed by the combined effects of nonlinear elastic deformation and time-dependent viscous resistance. Both models reproduced the measured monotonic stress–strain curves with high accuracy within the investigated quasi-static loading-speed range, demonstrating their suitability for describing the effective tensile response of the present hierarchical PAN nanofiber yarns. The strong consistency of the identified parameters further indicates that the two models are functionally equivalent for the present tensile dataset, although they differ in physical interpretation and structural representation. Overall, this work provides a compact constitutive framework for the mechanical characterization of hierarchical nanofiber yarns and may offer practical guidance for simplified model selection and parameter identification under monotonic tensile loading. It should be noted that the present models were established and validated only under monotonic tensile loading at selected quasi-static loading speeds. Therefore, the current parameters should be interpreted as effective constitutive parameters for the monotonic tensile response within the investigated experimental range. Cyclic loading–unloading behavior, creep, stress relaxation, fatigue damage, residual deformation, temperature-dependent mechanical behavior, and internal damage evolution were not addressed in this study and require further experimental validation and model extension. These aspects will be investigated in future work to further assess the generalizability and predictive capability of the proposed constitutive framework.

## Figures and Tables

**Table 1:** Goodness of fit of each model for high-strength nanofiber yarn at different strain rates.

Strain Rate	Model	R	$R^{2}$	RMSE
k=1/min	1	0.9998	0.9997	3.1178
	2	0.9998	0.9997	3.1178
k=10/min	1	0.9996	0.9991	6.0370
	2	0.9996	0.9991	6.0370

**Table 2:** Characteristic parameters of high-strength nanofiber yarn in two optimal models.

Model	η	$E_{1}$	$E_{2}$	b	C
1	77.09	/	94.66	−0.69	60.50
2	77.09	94.66	60.50	−0.69	/

**Table 3:** Comparison of the present approach with representative constitutive models reported in the literature.

Study	Material and Loading Condition	Model Type	Main Parameter Features
Chailleux and Davies [11,12]	Aramid/polyester fibers and yarns; tensile loading	Nonlinear viscoelastic–viscoplastic model	Elastic, viscous, and viscoplastic parameters
Liu et al. [13]	Textile yarns; relaxation	Generalized Maxwell-type model	Relaxation modulus and relaxation time
Ma et al. [15]	Synthetic fiber ropes; creep and recovery	Nonlinear creep-recovery model	Elastic, viscous, and creep parameters
Handelshauser et al. [16]	Collagen fibrils; tensile loading	Nonlinear Maxwell model	Nonlinear Maxwell-type parameters
This work	High-strength PAN nanofiber yarns; monotonic tensile loading	Modified nonlinear three-element and structural four-element models	Compact elastic, viscous, and nonlinear elastic parameters

## Data Availability

Data are contained within the article. Additional data are available from the corresponding author upon reasonable request.
